# Prevalence of human visceral leishmaniasis and its risk factors in Eastern Africa: a systematic review and meta-analysis

**DOI:** 10.3389/fpubh.2024.1488741

**Published:** 2024-11-21

**Authors:** Abebe Kassa Geto, Gete Berihun, Leykun Berhanu, Belay Desye, Chala Daba

**Affiliations:** ^1^Department of Nursing and Midwifery, Dessie Health Science College, Dessie, Ethiopia; ^2^Department of Environmental Health, College of Medicine and Health Sciences, Debre Markos University, Debre Markos, Ethiopia; ^3^Department of Environmental Health, College of Medicine and Health Sciences, Wollo University, Dessie, Ethiopia; ^4^National Centre for Epidemiology and Population Health, The Australian National University, Canberra, ACT, Australia

**Keywords:** Eastern Africa, human, outdoor sleeping, termite hill, visceral leishmaniasis

## Abstract

**Introduction:**

Visceral Leishmaniasis, also known as kala-azar, is a potentially fatal, neglected tropical disease caused by the protozoan parasite *Leishmania* and transmitted through infected sandflies. It is one of the major global public health problems and contributors to economic crisis among people. Though different studies investigated human visceral leishmaniasis in Eastern Africa, the findings were inconsistent and inconclusive enough, and there is no representative data on this devastating public health concern. Therefore, this systematic review and meta-analysis aimed to determine the pooled prevalence and risk factors associated with human visceral leishmaniasis in Eastern Africa.

**Methods:**

The Preferred Reporting Items for Systematic Reviews and Meta-Analysis (PRISMA 2020) guidelines were followed for this study. Databases such as PubMed/MEDLINE, CINAHL, LIVIVO, African Journals Online, African Index Medicus (AIM), HINARI, Science Direct, Web of Science, Cochrane Library, Google Scholar, Semantic Scholar, and Google were used to retrieve all the relevant articles. The search was carried out from 23 May 2024 to 17 July 2024. Data were analyzed using STATA 17 software to determine the pooled prevalence of human visceral leishmaniasis with a 95% confidence interval using a random-effects model.

**Result:**

In this meta-analysis, thirty-nine articles with 40,367 study participants were included. The overall pooled prevalence of human visceral leishmaniasis in Eastern Africa was 26.16% [95%; CI: 19.96, 32.36%; I^2^ = 99.67%; *p* = 0.00]. Gender, age, family size, presence of termite hill/mound, presence of cattle/domestic animals, outdoor sleeping, presence of VL infected family member/s, and presence of water source/pathway near home were the risk factors significantly associated with human visceral leishmaniasis.

**Conclusion:**

The recorded pooled prevalence of human visceral leishmaniasis in Eastern Africa underscores the urgent need for comprehensive intervention strategies. This includes rigorous health education for residents, covering the disease’s cause, transmission, vector breeding sites, and prevention mechanisms.

## Introduction

Visceral leishmaniasis (VL), known as kala-azar, is a potentially fatal, neglected tropical disease caused by the protozoan parasite *Leishmania*. Transmitted through the bite of infected sandflies, VL primarily affects impoverished communities (1–4). VL is characterized by severe symptoms including fever, weight loss, fatigue, weakness, loss of appetite, enlarged liver and spleen, anemia, and swollen lymph nodes (2, 5). It is the most serious form of leishmaniasis, posing a life-threatening risk if left untreated (6). The parasite, *Leishmania donovani*, thrives in humans and sandflies, making human populations in Asia and Eastern Africa particularly vulnerable (1, 4, 7, 8). This neglected disease disproportionately impacts the poorest communities, highlighting the urgent need for improved prevention, diagnosis, and treatment strategies to combat its devastating effects (5).

VL is endemic in 80 countries with an estimated annual global incidence of 50,000–90,000 worldwide (9, 10). According to the 2022 World Health Organization (WHO) report, about 85% of global VL cases were reported from seven countries: Brazil, Ethiopia, India, Kenya, Somalia, South Sudan and Sudan (10). The 2017 World Health Organization (WHO) report revealed significant burden of VL in Eastern Africa, with South Sudan reporting the highest number of cases (3474) (11) followed by Sudan with 2902 (12), Ethiopia with 2,141 (13), and Somalia 1,166 cases (14). The disease has also been reported in Kenya, Uganda, and Eritrea (15–17). This trend continued in 2022, with Eastern Africa accounting for a staggering 73% of global VL cases. Alarmingly, half of these cases occurred in children under the age of 15, underscoring the devastating impact of this neglected disease on young lives (18).

A comprehensive global analysis of blood donor data revealed a pooled prevalence of visceral leishmaniasis at 7% (19). Different systematic reviews and meta-analysis conducted on human VL in Iran revealed that the pooled prevalence was 2–3% (2, 20). Moreover, systematic reviews and meta-analysis of studies conducted in Ethiopia, where VL is endemic, revealed a pooled prevalence of the disease ranging from 9.44 to 21%, 9.44% (21) to 21% (22), underscoring the high burden of VL within the country. VL imposes a significant economic burden on affected families in different countries. The costs associated with healthcare, including informal payments for accessing providers, diagnostics and medication, and transport costs for multiple visits can be substantial. These high direct expenses often force individuals to adopt coping strategies like selling or renting assets and taking out loans, which further exacerbating their financial hardship (23–25). Despite the goal of universal health coverage, remote areas with endemic visceral leishmaniasis (VL) face significant obstacles. Their weak and under-resourced health systems struggle to integrate complex VL diagnostic and treatment services into their limited basic healthcare packages. This challenge is further compounded by factors like economically driven migration or massive population displacements during conflicts, exacerbating the burden of the disease (26). Despite being a neglected tropical disease, visceral leishmaniasis (VL) has been prioritized for elimination by 2030 under the Sustainable Development Goals (SDGs), specifically Target 3.3 (27).

Preventing visceral leishmaniasis in Eastern Africa is a complex challenge due to the numerous interconnected risk factors associated with the disease. Factors such as age, sex, environmental conditions like the presence of certain trees or termite mounds, animal ownership such as cattle and dogs, living conditions, awareness levels, occupation, and education all contribute to the disease’s spread. To effectively address the impact and severity of VL, a multi-faceted approach is crucial, considering the interplay of these various risk factors (28–42). Behavioral factors, such as sleeping outdoors, neglecting bed nets, and inadequate insecticide spraying, along with housing conditions like wall type, have been identified as significant risk factors for VL in various studies (29, 31, 32, 34, 36–38, 43–45).

Although many primary studies have been carried out on human visceral leishmaniasis among the countries of Eastern Africa, the findings have not been consistent with the prevalence ranging from 1.8 to 84.8%. Moreover, the findings were not conclusive, and these could hamper the assessments of ongoing intervention efforts and activities (46, 47). Moreover, no study provides evidence of the pooled prevalence of human visceral leishmaniasis in Eastern Africa. Therefore, this systematic review and meta-analysis aimed to estimate the pooled prevalence of human visceral leishmaniasis and identify its risk factors in Eastern Africa. This systematic review and meta-analysis underscore the critical need for prioritizing primary prevention of human visceral leishmaniasis in Eastern Africa. This evidence-based information will be invaluable for policymakers, healthcare planners, and other stakeholders in developing and implementing comprehensive strategies to prevent, control, and mitigate the impact of VL. These insights can contribute to a future where the burden of this neglected tropical disease is significantly reduced in Eastern Africa.

## Materials and methods

### Study registration

The protocol of this systematic review and meta-analysis was registered on the International Prospective Register of Systematic Reviews (PROSPERO) database under registration number CRD42023427719.

### Search strategy

The search was carried out from 23 May 2024 to 17 July 2024 by three independent authors (AKG, CD, and LB). The identified articles were imported into Endnote version 8 software, where duplicate entries were removed. All the relevant articles were selected based on the Preferred Reporting Items for Systematic Reviews and Meta-Analysis (PRISMA) guidelines (48).

In this systematic review and meta-analysis, published and unpublished studies were searched from different electronic databases such as PubMed/MEDLINE, Science Direct, Cochrane Library, LIVIVO, CINAHL, African Journals Online, Web of Science, African Index Medicus (AIM), HINARI, Semantic Scholar, Google, and Google Scholar. In addition, gray literature was also identified from digital libraries and repositories of different universities. The search was carried out using the following keywords: “prevalence,” “epidemiology,” “Visceral Leishmaniasis,” “Kala-Azar,” “Kala Azar “, “Black Fever “, “Black Disease,” Dumdum Fever *, “Black Sickness,” “*Leishmania infantum*,” “*Leishmania donovani*,” Human Leishmaniosis,” “Human Leishmaniasis,” “Leishmaniasis,” “Leishmaniases,” “associated factor*,” “risk factor*,” determinant*, predictor*, cause*, Burundi, Comoros, Djibouti, Ethiopia, Eritrea, Kenya, Rwanda, Seychelles, Somalia, South Sudan, Sudan, Tanzania, and Uganda. Using the appropriate Boolean operators: “AND” or “OR,” all keywords were combined.

### Inclusion criteria

This systematic review and meta-analysis included all published and unpublished studies conducted from January 1982 to July 2024 in countries of Eastern Africa that investigated human visceral leishmaniasis. The studies were conducted both in community and institutional settings and employed cross-sectional, cohort, and case–control designs. Only studies reported in English were included.

#### Population

All studies conducted on all groups of human population were included.

#### Exposure

People infected with human VL.

#### Comparisons

People that were not infected with human VL.

### Exclusion criteria

This systematic review and meta-analysis excluded studies that lacked full text, were unidentified, were abstracts, editorials, irretrievable, letters, or did not report human visceral leishmaniasis as the primary outcome.

### Measurement of the outcome

This study aimed to determine the pooled prevalence of human visceral leishmaniasis in countries of Eastern Africa. This was calculated by dividing the number of study participants with VL by the total sample size and multiplying by 100. Additionally, the systematic review and meta-analysis investigated factors associated with VL.

### Operational definition

Visceral Leishmaniasis: Also known as kala-azar, is a potentially fatal, neglected tropical disease caused by the protozoan parasite *Leishmania*, transmitted through infected sandflies and characterized by severe symptoms including fever, weight loss, fatigue, weakness, loss of appetite, enlarged liver and spleen, anemia, and swollen lymph nodes (1–5).

### Data extraction procedure

A standard data extraction template consisting of several details such as author name, year of publication, country, study setting, study design, method of VL detection, sample size, and prevalence was prepared. Duplicate articles were removed after the relevant articles for inclusion were carefully screened. Three independent authors (AKG, BD, and LB) were involved to undertake the required data extraction activities.

### Quality assessment

Using the Joana Brigg Institute (JBI) checklist of critical appraisal for cross-sectional studies, the quality of each article was critically evaluated (49) (Supplementary material). With scores measured on a scale of 100%, the quality of each article was independently assessed by three authors (AKG, CD, and GB). For further analysis, articles having a quality score of above 50% were included (50, 51). A mean score was calculated from the evaluation results of all the reviewers for an agreement in case of any differences when undertaking the quality assessment.

### Study selection

**Figure 1:**
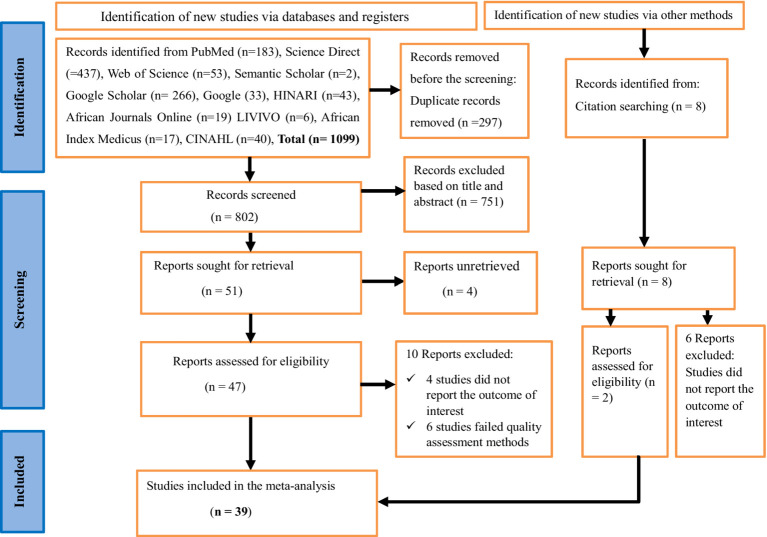
A PRISMA flow chart showing study selection for systematic review and meta-analysis of the prevalence of human visceral leishmaniasis and its risk factors in Eastern Africa, 2024.

Overall, 1,107 studies were identified from an electronic database and reference searching. An Endnote 8 reference manager was used. Two hundred and ninety-seven duplicated articles were removed. The total number of articles excluded based on their titles and abstracts due to the failure to meet the inclusion criteria was 751. Moreover, ten articles were removed as they did not report the outcome of interest and six articles were excluded as they failed to meet quality assessment methods. In this meta-analysis, 39 full-text articles were included to estimate the pooled prevalence of human VL in Eastern Africa by following the PRISMA 2020 guideline (Figure 1).

### Statistical analysis procedures

After the data were extracted, analysis was made using STATA (Corporation, College Station, Texas, United States) version 17 software after the extracted data were successfully imported in to it. Heterogeneity within the included studies was assessed using the Higgs I^2^ test, with values of 75, 50, and 25% showing high, moderate, and low levels of heterogeneity, respectively (52). With a 95% confidence interval, a restricted maximum-likelihood (53) method of random-effects model was used to determine the pooled prevalence of human visceral leishmaniasis in Eastern Africa. The odds ratio was computed to show the strength of the association between human VL (the outcome variable) and its risk factors.

The pooled prevalence of human visceral leishmaniasis was presented using a forest plot. To determine the influence of an individual study on the pooled prevalence estimate of VL, sensitivity analysis was performed. Sub-group analysis was also done to identify the possible sources of heterogeneity based on the year of publication (before 2016 and 2016 and after), country category (Ethiopia, Kenya, Sudan, and other countries), study setting (healthcare facility-based and community-based), study design (cross-sectional, case–control and cohort), and sample size category (lower than 1,000, and 1,000 and above). Additionally, a funnel plot and Egger’s test were used to determine the presence of potential publication bias (54).

## Results

### Characteristics of the included studies

In this meta-analysis, twenty-six cross-sectional (28–31, 33, 34, 36–38, 40–45, 47, 55–64), eight cohort (46, 65–71), and five case–control (32, 35, 39, 72) studies with a total of 40,367 study subjects were included. The highest prevalence of human VL was 84.8% (46), and the lowest prevalence was 1.8% (47) among the included studies. Regarding the country in which the studies were conducted, twenty-four were in Ethiopia (3, 29–34, 37, 41–47, 56–58, 64–66, 69–71), seven studies were in Kenya (28, 35, 38, 60, 61, 63, 72), five studies were in Sudan (36, 39, 55, 59, 62), two studies were in Uganda (40, 68), and the other study was conducted in Somalia (67). Based on study setting, twenty-two of the included studies (28–31, 33–41, 43, 47, 56–60, 63, 72) were community-based and the remaining fifteen studies (32, 42, 44–46, 55, 61, 62, 65–71) were healthcare facility-based. Regarding the diagnostic method/s of VL, twenty-five studies used serological examination (3, 29–32, 34, 35, 37, 39, 40, 44, 45, 47, 55, 56, 60, 61, 63–67, 70–72), five studies used immunological test (33, 41, 57–59), six studies (28, 36, 38, 43, 46, 69) unspecified the diagnostic method/s used, and the remaining three studies used molecular diagnosis (62), a combination of serological and molecular diagnosis (42), and a combination of serological and parasitological examination (68). Regarding the year of publication, twenty-three of the included studies (28–31, 33–36, 38, 41, 42, 44–47, 56, 61, 62, 64, 66, 70–72) were published in 2016 and after, and the remaining 16 studies (3, 32, 37, 39, 40, 43, 55, 57–60, 63, 65, 67–69) were published before 2016. Based on the sample size, thirty-two studies had a sample size of lower than1000 (28–32, 34–46, 55–63, 65, 67, 72), and the remaining seven studies had a sample size of 1,000 and above (33, 47, 66, 68–71) (Table 1).

**Table 1:** Characteristics of the included studies to determine the pooled prevalence of human VL in Eastern Africa, 2024.

Authors	Publication year	Country	Study setting	Study design	Method/s of VL diagnosis	Sample size	Prevalence (%)	Quality score (%)
Abdullahi et al. (28)	2022	Kenya	Community-based	Cross-sectional	Unspecified	360	21.7	87.5
Dulacha et al. (35)	2019	Kenya	Community-based	Case–control	Serological Examination	231	33.3	90
Ho et al. (60)	1982	Kenya	Community-based	Cross-sectional	Serological Examination	267	3.7	62.5
Kanyina (61)	2020	Kenya	Healthcare facility-based	Cross-sectional	Serological Examination	433	31.4	100
Lotukoi (38)	2020	Kenya	Community-based	Cross-sectional	Unspecified	341	49.3	62.5
Ryan et al. (63)	2006	Kenya	Community-based	Cross-sectional	Serological Examination	489	31.5	62.5
van Dijk et al. (72)	2023	Kenya	Community-based	Case–control	Serological Examination	86	41.9	90
Abdalla et al. (55)	2024	Sudan	Healthcare facility-based	Cross-sectional	Serological Examination	69	82.6	75
El-Safi et al. (59)	2002	Sudan	Community-based	Cross-sectional	Immunological Test	947	20.9	62.5
Ibrahim et al. (36)	2024	Sudan	Community-based	Cross-sectional	Unspecified	500	27.6	62.5
Mohamed et al. (62)	2019	Sudan	Healthcare facility-based	Cross-sectional	Molecular Diagnosis	95	36.8	100
Nackers et al. (39)	2015	Sudan	Community-based	Case–control	Serological Examination	999	19.8	80
Odoch and Olobo (40)	2013	Uganda	Community-based	Cross-sectional	Serological Examination	285	17.2	75
Mueller et al. (68)	2009	Uganda	Healthcare facility-based	Cohort	Serological and Parasitological Examination	3,485	54.6	54.5
Abera et al. (56)	2016	Ethiopia	Community-based	Cross-sectional	Serological Examination	275	7.3	62.5
Ayalew and Abere (46)	2019	Ethiopia	Healthcare facility-based	Cohort	Unspecified	369	84.8	54.5
Ayehu et al. (30)	2018	Ethiopia	Community-based	Cross-sectional	Serological Examination	185	7.6	87.5
Azene et al. (31)	2017	Ethiopia	Community-based	Cross-sectional	Serological Examination	398	6.5	62.5
Bantie et al. (32)	2014	Ethiopia	Healthcare facility-based	Case–control	Serological Examination	545	20	70
Bejano et al. (33)	2021	Ethiopia	Community-based	Cross-sectional	Immunological Test	1,197	6	100
Bsrat et al. (34)	2018	Ethiopia	Community-based	Cross-sectional	Serological Examination	329	8.81	100
Custodio et al. (43)	2012	Ethiopia	Community-based	Cross-sectional	Unspecified	565	9.91	87.5
Gize et al. (66)	2020	Ethiopia	Healthcare facility-based	Cohort	Serological Examination	9,299	21	72.7
Ismail et al. (44)	2023	Ethiopia	Healthcare facility-based	Cross-sectional	Serological Examination	187	9.63	100
Lemma et al. (37)	2015	Ethiopia	Community-based	Cross-sectional	Serological Examination	359	12.5	100
Melkie et al. (45)	2023	Ethiopia	Healthcare facility-based	Cross-sectional	Serological Examination	205	15.6	87.5
Tadese et al. (41)	2019	Ethiopia	Community-based	Cross-sectional	Immunological Test	650	9.08	100
Terefe et al. (69)	2015	Ethiopia	Healthcare facility-based	Cohort	Unspecified	1,270	29.4	63.6
van Griensven et al. (42)	2019	Ethiopia	Healthcare facility-based	Cross-sectional	Serological and Molecular Test	511	9.6	100
Yimer et al. (71)	2022	Ethiopia	Healthcare facility-based	Cohort	Serological Examination	2,703	32.4	81.8
Alebie et al. (29)	2019	Ethiopia	Community-based	Cross-sectional	Serological Examination	361	15.8	100
Ali and Ashford (57)	1993	Ethiopia	Community-based	Cross-sectional	Immunological Test	728	36.4	62.5
Ali et al. (58)	2004	Ethiopia	Community-based	Cross-sectional	Immunological Test	767	38.3	62.5
Diro et al. (65)	2015	Ethiopia	Healthcare facility-based	Cohort	Serological Examination	928	33.2	54.5
Wondimeneh et al. (70)	2014	Ethiopia	Healthcare facility-based	Cohort	Serological Examination	7,161	39.1	72.7
Bekele et al. (47)	2018	Ethiopia	Community-based	Cross-sectional	Serological Examination	1,681	1.8	100
Ketema et al. (64)	2022	Ethiopia	Community-based	Cross-sectional	Serological Examination	432	7.6	100
Yared et al. (3)	2014	Ethiopia	Community-based	Case–control	Serological Examination	283	31.8	90
Marlet et al. (67)	2003	Somalia	Healthcare facility-based	Cohort	Serological Examination	392	58.7	54.5

### Meta-analysis

#### Pooled prevalence of human visceral leishmaniasis

**Figure 2:**
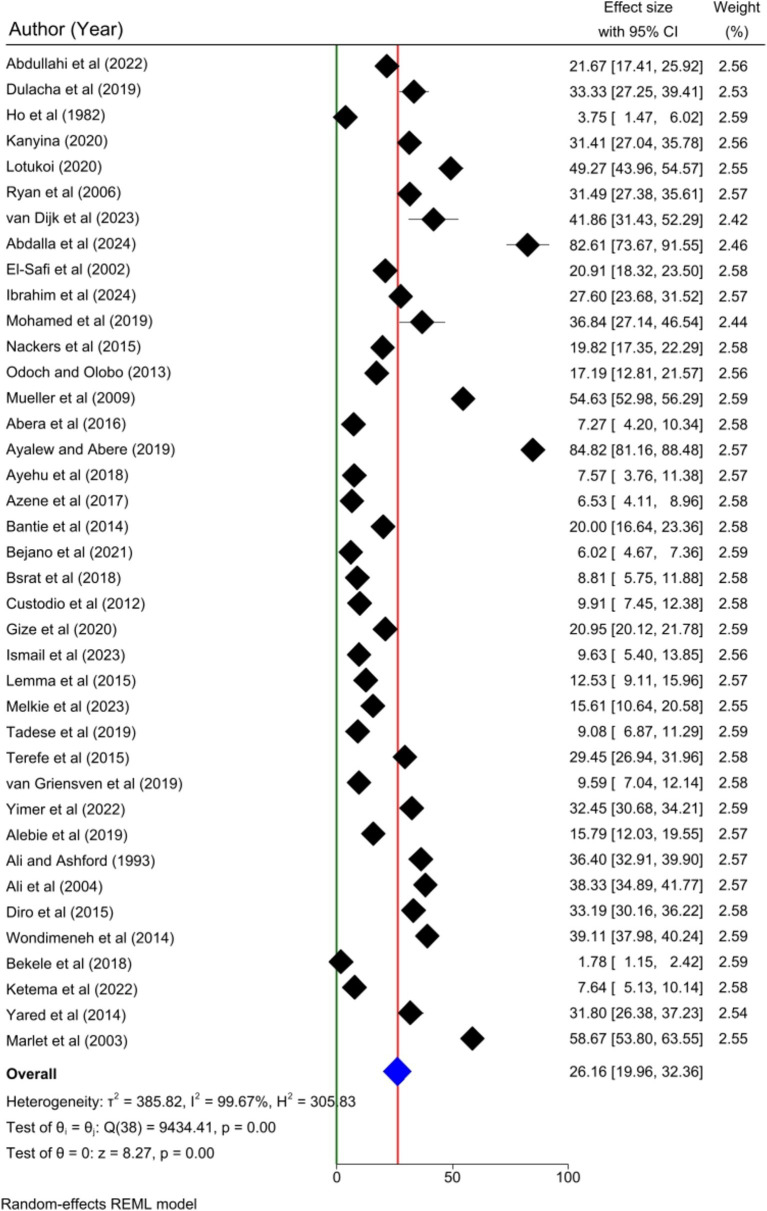
Forest plot showing the pooled prevalence of human VL in Eastern Africa, 2024.

Thirty-nine articles were included to determine the pooled prevalence of human visceral leishmaniasis in this meta-analysis. The pooled prevalence of human visceral leishmaniasis in Eastern Africa was 26.16% [95%; CI: 19.96, 32.36%; I^2^ = 99.67%; *p* = 0.00]. A random effects model was employed to estimate the pooled prevalence of human VL (Figure 2).

#### Test for publication bias

**Figure 3:**
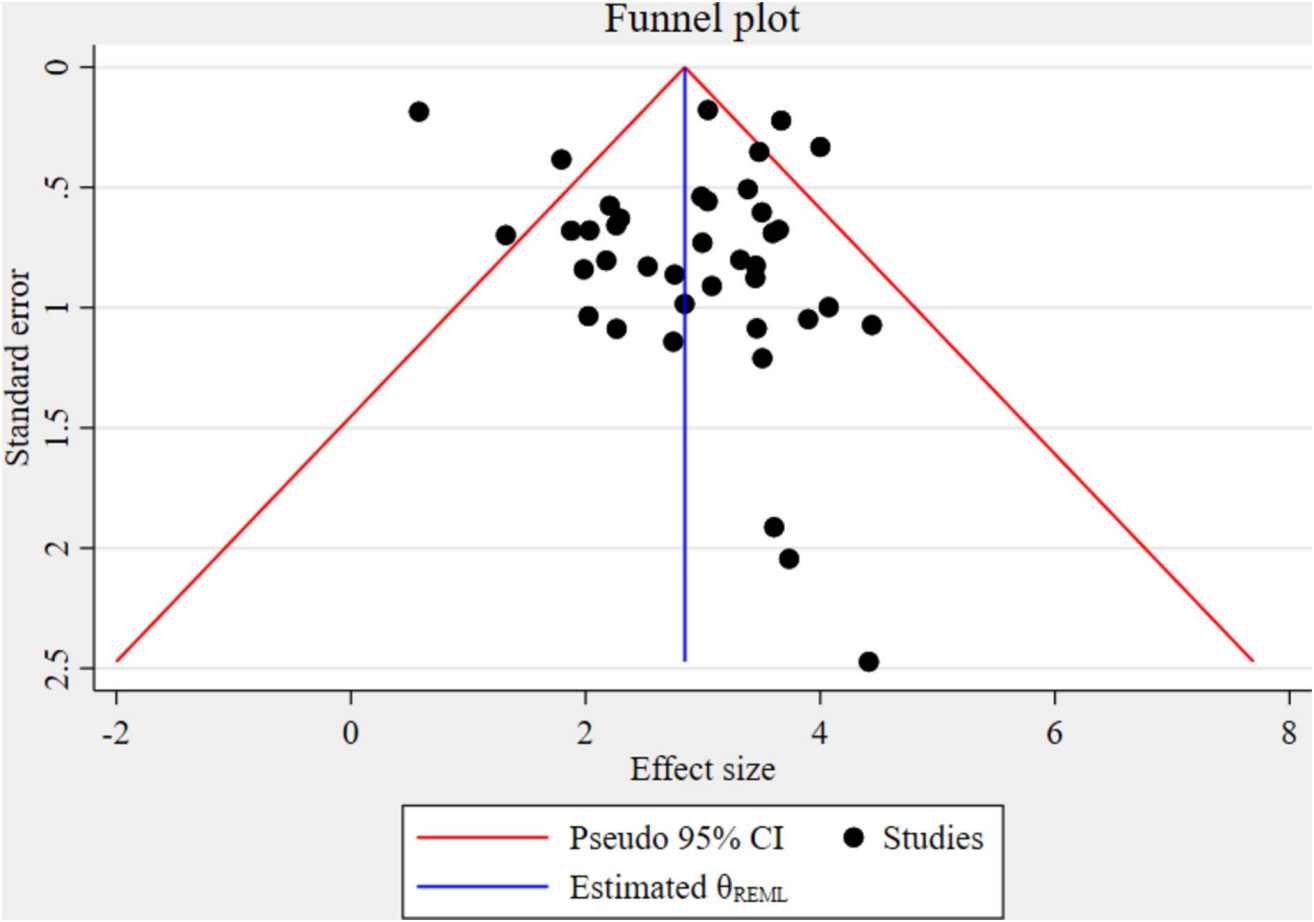
A Funnel plot to test the publication bias of the included studies in the meta-analysis.

**Figure 4:**
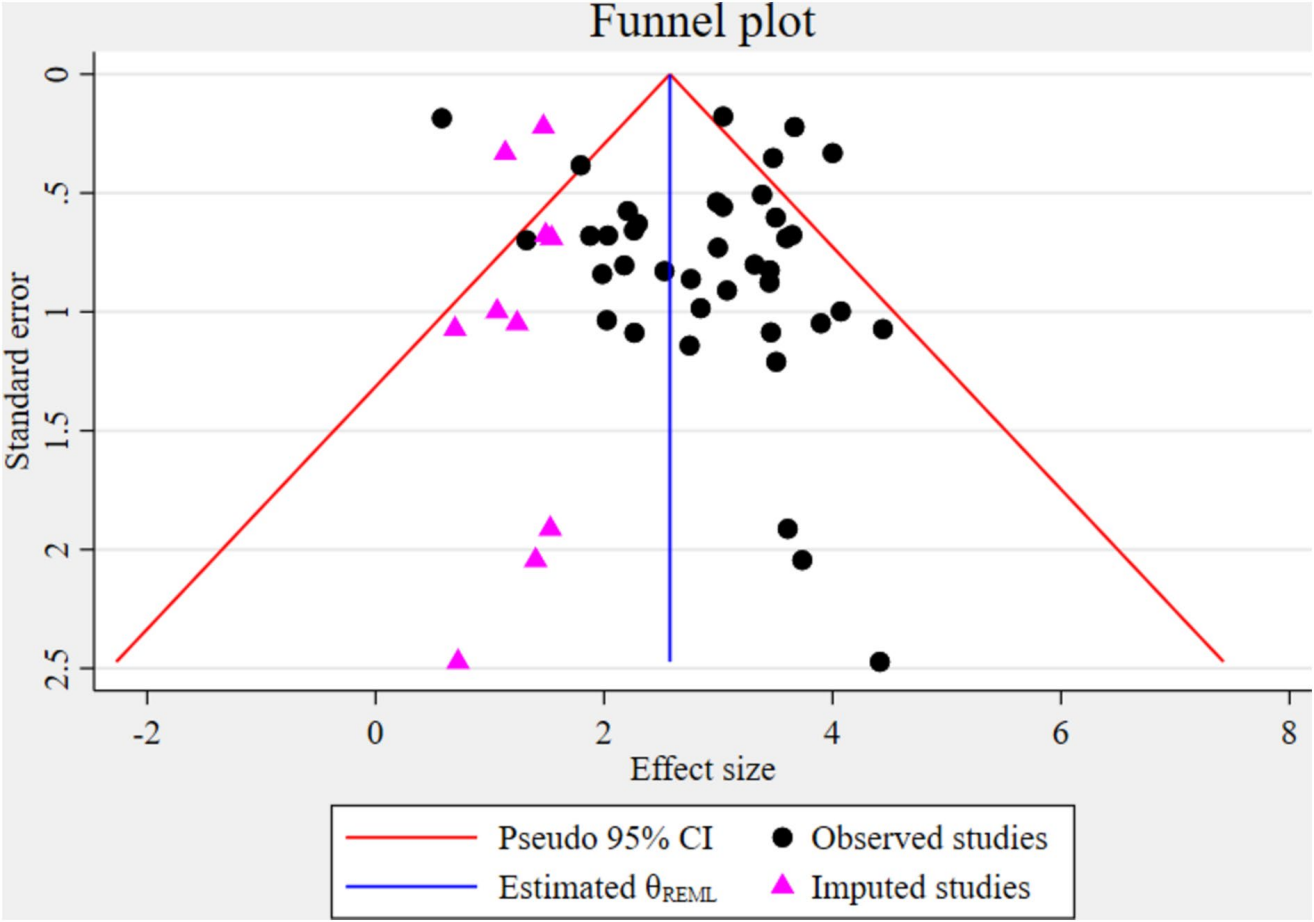
Funnel plot displaying the result of the simulated meta-analysis.

The funnel plot indicated significant publication bias (Figure 3). Statistically, Eggers’s test result also depicted statistically significant publication bias (small studies effect) (*p* = 0.028). A trim and fill analysis was conducted to identify the source of publication bias, resulting in a notable variation in the adjusted point estimate of the pooled odds ratio (OR = 2.58; 95% CI: 2.26–2.89), compared to the initial or observed point estimate (OR = 2.85; 95% CI: 2.52–3.18) (Figure 4).

### Sensitivity analysis

**Figure 5:**
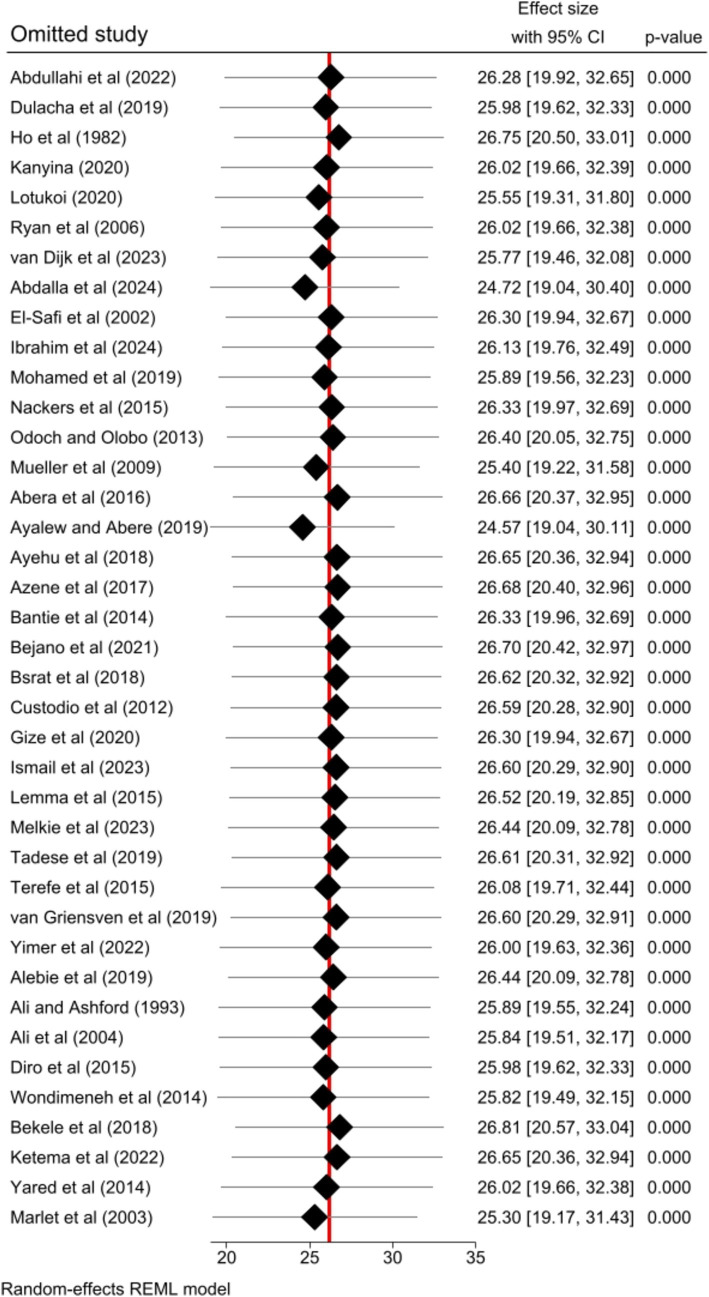
Sensitivity analysis result of the included studies for the pooled prevalence of human VL in Eastern Africa, 2024.

The impact of individual studies on the pooled estimate of human VL was successfully evaluated by performing a sensitivity analysis. The finding revealed that none of the included studies affected the pooled estimate (Figure 5).

### Subgroup analysis

**Figure 6:**
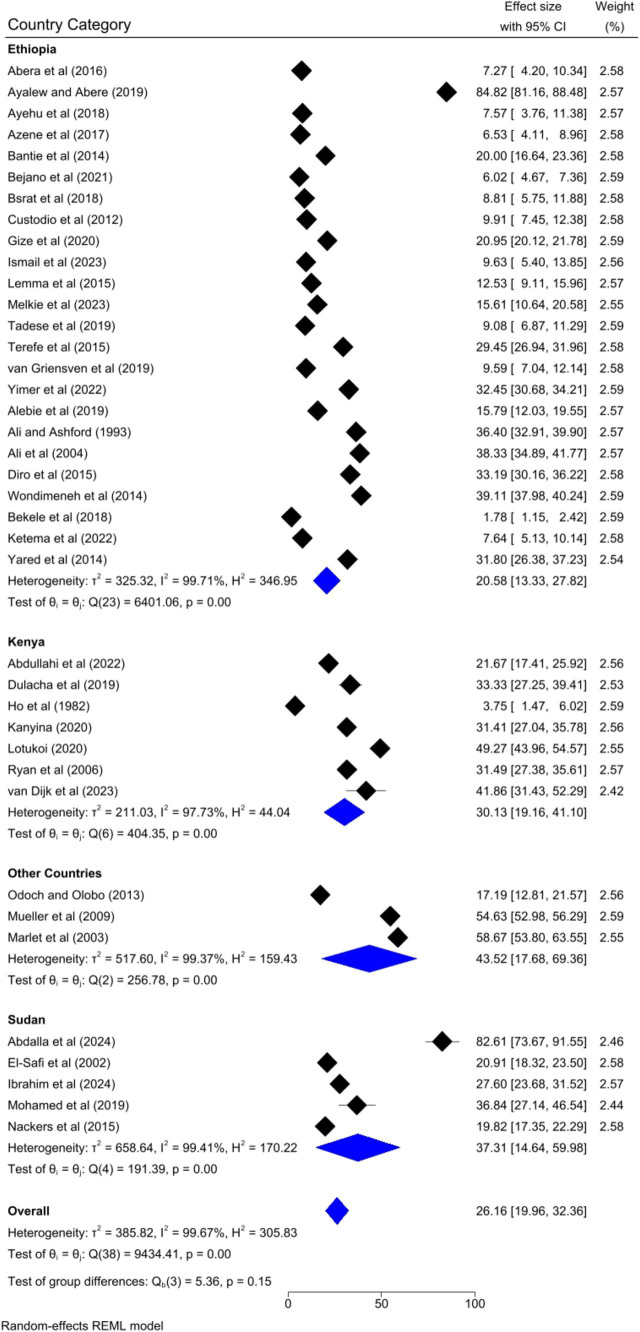
Subgroup analysis by country category.

**Figure 7:**
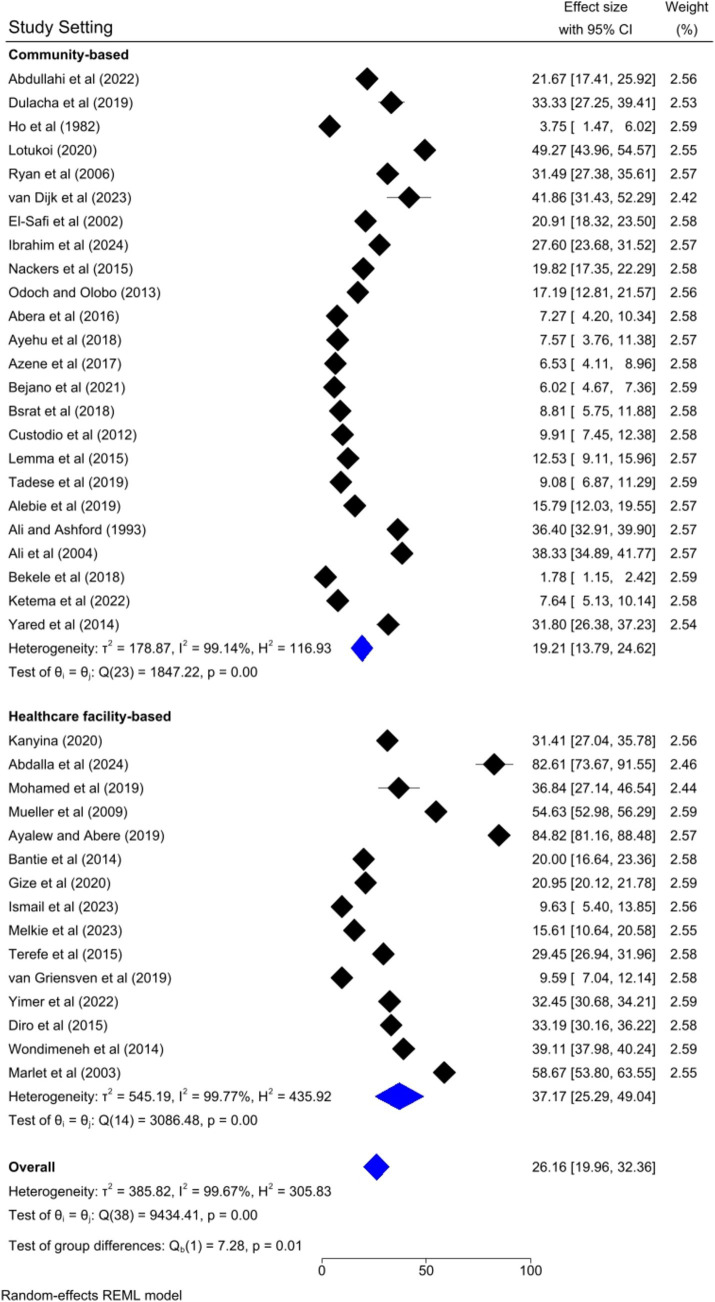
Subgroup analysis by study setting.

**Figure 8:**
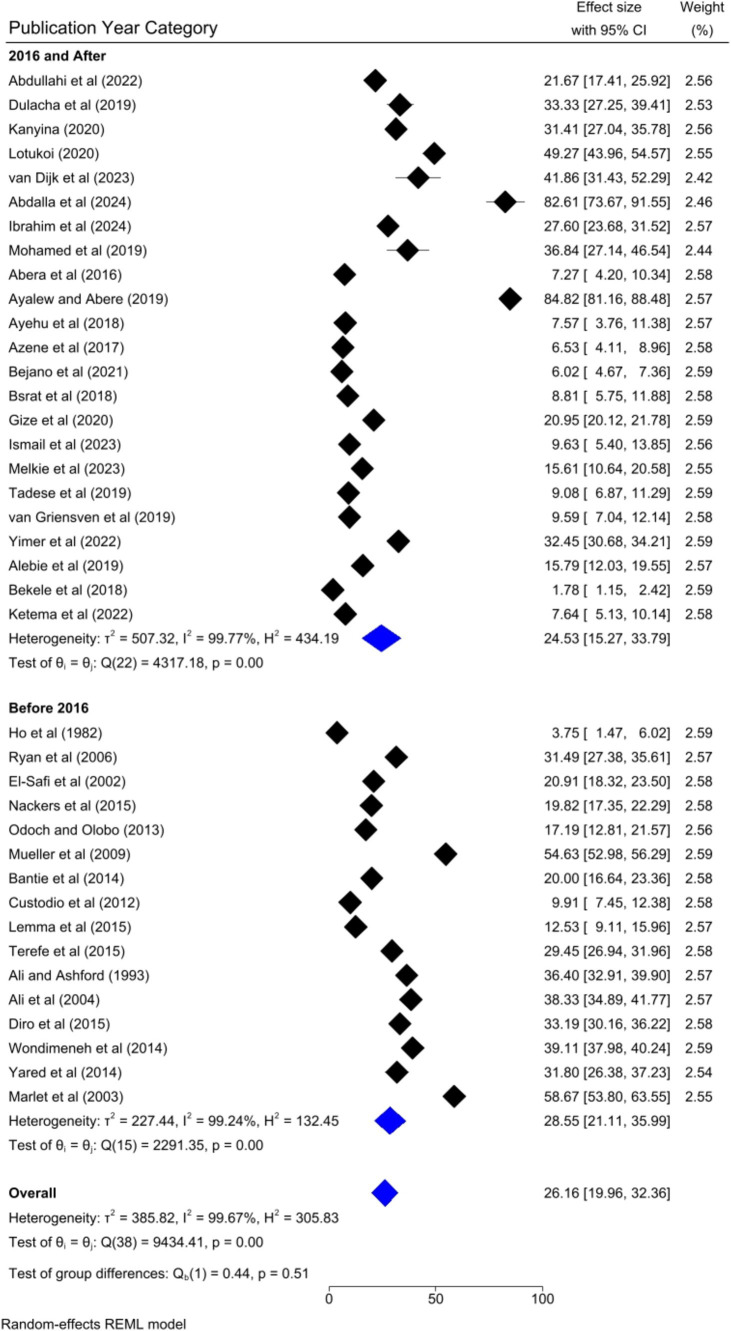
Subgroup analysis by publication year category.

Based on the country category in which the studies were conducted, the highest human VL pooled prevalence was registered in studies conducted in other countries (i.e., Uganda and Somalia) [43.52, 95% CI: 17.68–69.36%] as compared to studies conducted in Sudan [37.31, 95% CI: 14.64–59.98%], Kenya [30.13, 95% CI: 9.161–41.10%], and Ethiopia [20.58, 95% CI: 13.33–27.82%] (Figure 6). Based on the study setting in which the studies were conducted, the highest pooled prevalence of human VL was recorded among studies conducted at the healthcare facility [37.17, 95% CI: 25.29–49.04%], compared to studies conducted in the community [19.21, 95% CI: 13.79–24.62%] (Figure 7). Regarding the articles’ publication year category, the highest pooled human VL was observed among studies published before 2016 [28.55, 95% CI: 21.11–35.99%] as compared to those studies published in 2016 and after [24.53, 95% CI: 15.27–33.79%] (Figure 8).

**Figure 9:**
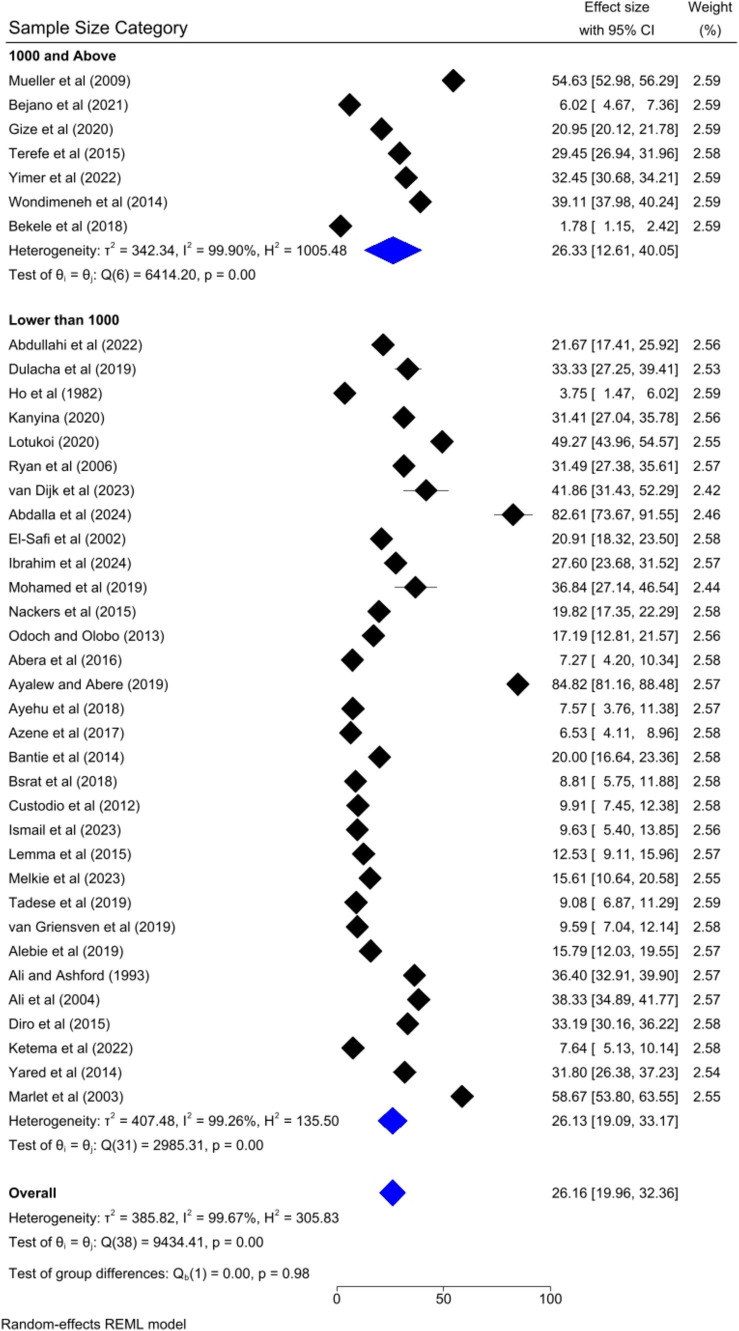
Subgroup analysis by sample size category.

**Figure 10:**
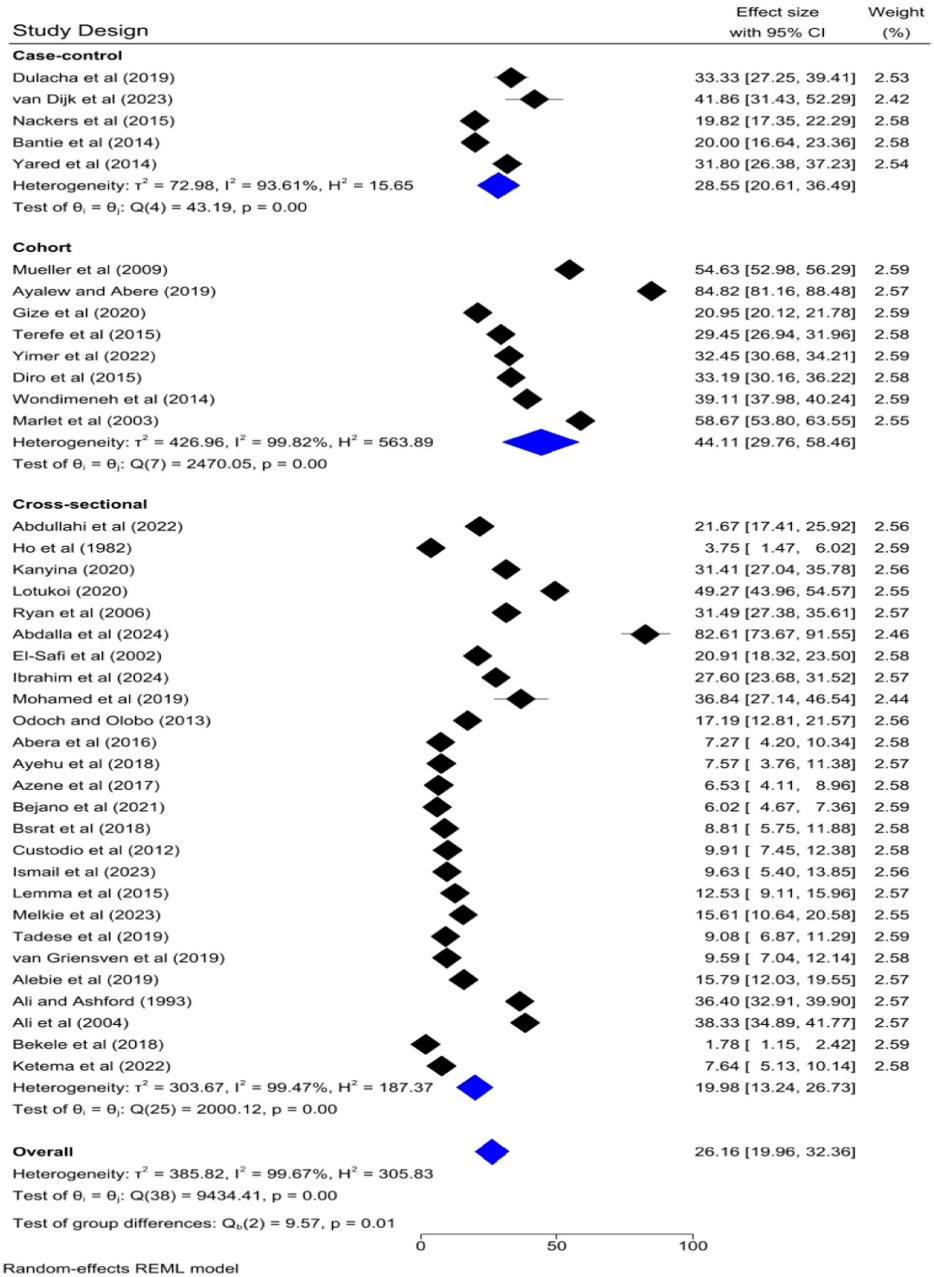
Subgroup analysis by study design.

The pooled prevalence of VL was slightly higher among studies that had a sample size of 1,000 and above [26.33, 95% CI: 12.61–40.05%] as compared to studies that had a sample size of lower than 1,000 [26.13, 95% CI: 19.09–33.17%] (Figure 9). Regarding the study design, the highest pooled prevalence of VL was registered among cohort studies [44.11, 95% CI: 29.76–58.46] followed by case–control [28.55, 95% CI: 20.61–36.49%] and cross-sectional studies [19.98, 95% CI: 13.24–26.73%] (Figure 10).

### Meta-regression

To identify the source of heterogeneity by considering, country category, study setting, study design, publication year category, and sample size category as factors, a univariate meta-regression analysis was performed. However, statistical significance was demonstrated by two of these variables (Table 2).

**Table 2:** A univariate meta-regression analysis to pinpoint the factors associated with the heterogeneity of human VL pooled prevalence in Eastern Africa, 2024.

Variables	Coefficient	Standard error	*p*-value
Country category	−0.2243004	0.1395502	0.118
Study setting	−0.9356682	0.3529824	0.012*
Study design	−0.4648124	0.3325146	0.171
Publication year category	−0.4681677	0.2742282	0.097
Sample size category	0.7304525	0.3258774	0.032*

* Indicate the statistical significance of the variables on heterogeneity.

### Risk factors associated with human visceral leishmaniasis in Eastern Africa

Twenty-three factors were repeatedly presented in the included articles of this meta-analysis. The factors were gender, age (< 15 years), educational status (primary education, secondary education, university/college education), family size >5, occupation (cattle keeping), house wall made from (mud and wood, mud and stone), house wall type (cracked), use of bed net, presence of acacia tree, presence of termite hill/mound, presence of cattle/domestic animal, presence of animal shed, presence of dogs, outdoor sleeping, sleeping under Balanites/acacia tree, knowing sign and symptoms of VL, knowing transmission of VL, presence of VL- infected family member/s, travel history to endemic areas, and presence of water source/pathway near home (Table 3).

**Table 3:** The pooled odds ratio for risk factors associated with human VL in Eastern Africa, 2024.

Listed variables		Number of study participants	Number of studies included	Pooled Odds Ratio (95% CI)	Heterogeneity	
					I^2^ (%)	*p*-value
Gender (male)		6,651	13	1.68 (1.33–2.03)*	0.0	0.450
Age (<15 Years)		1,235	3	2.09 (1.13–3.04)*	0.0	0.457
Educational status	Primary education	841	2	2.09 (0.78–3.40)	0.0	0.640
	Secondary education	841	2	0.98 (−0.62–2.59)	0.0	0.562
	University/College education	841	2	1.05 (−0.35–2.44)	50.7	0.154
Occupation (cattle keeping)		1,395	3	1.35 (0.29–2.42)	4.5	0.351
Family size (>5)		663	2	2.83 (1.03–4.63)*	43.5	0.183
House wall made from	Mud and wood	1,304	3	1.02 (0.37–1.67)	0.0	0.400
	Mud and stone	759	2	1.22 (−0.75–3.18)	0.0	0.950
House wall type (cracked)		715	2	1.97 (0.06–3.88)	0.0	0.372
Use of bed net (no)		1,599	4	1.40 (1.32–2.12)	60.1	0.057
Presence of acacia tree (yes)		3,223	7	1.60 (0.71–2.49)	70.0	0.003
Presence of termite hill/mound (yes)		2,327	6	2.68 (1.51–3.85)*	0.0	0.478
Presence of cattle/domestic animal (yes)		1,560	4	2.21 (1.02–3.40)*	0.0	0.681
Presence of animal shed (yes)		1,190	3	0.88 (−0.33–2.09)	16.5	0.302
Presence of dogs (yes)		3,411	5	2.23 (0.73–3.73)	69.6	0.011
Sleeping outdoor (yes)		3,085	8	3.76 (2.26–5.25)*	0.0	0.793
Sleeping under Balanites/acacia tree (yes)		1,015	3	0.58 (−0.19–1.36)	30.0	0.240
Knowing sign and symptoms of VL (no)		1,257	3	0.28 (−0.28–0.83)	30.6	0.237
Knowing transmission of VL (no)		1,083	3	1.90 (−0.73–4.52)	70.9	0.032
Presence of VL- infected family member/s (yes)		1,035	3	3.59 (1.94–5.24)*	0.0	0.435
Travel history to endemic areas (yes)		663	2	1.54 (0.11–2.97)	66.7	0.083
Presence of water source/pathway near home (yes)		792	2	4.29 (1.89–6.70)*	0.0	0.771

* Indicates a significant association of variables with the pooled prevalence of human VL.

The association between gender and human VL among the thirteen studies (29, 32–35, 38–41, 43, 45, 64, 72) has been assessed. The result showed a significant association in nine of the included studies. According to this meta-analysis, the odds of VL infection were 68% higher among males compared to females [POR = 1.68; 95% CI: 1.33–2.03]. The association between age below 15 years and VL among the three studies (29, 32, 34) has been determined. The result showed a significant association in two of the studies. According to the results of the random effect meta-analysis, the odds of VL infection were 2.09 times higher among people aged below 15 years compared to people aged 15 years and above [POR = 2.09; 95% CI: 1.13–3.04]. Two studies (35, 64) were considered to determine the association between family size >5 and VL. One of them was significantly associated with the outcome of interest. The odds of VL infection were 2.83 times higher among people who had a family of more than five as compared to their counterparts [POR = 2.83; 95% CI: 1.03–4.63].

Based on the findings of six studies (28, 29, 31, 32, 35, 64) the association between VL and the presence of termite hill/mound was assessed. In five of these studies, a positive association was found. According to the results of this meta-analysis, the odds of VL infection were 2.68 times higher among people who had termite hill/mound near their home compared to their counterparts [POR = 2.68; 95% CI: 1.51–3.85] (Table 3). The link between the presence of cattle/domestic animals and the outcome variable VL was assessed with four articles (30–32, 64). Their link was significant in three of the included studies. This finding revealed that the odds of VL infection were 2.21 times higher among people who had cattle/domestic animals compared to their counterparts [POR = 2.21; 95% CI: 1.02–3.40].

Eight articles (29, 31, 32, 34, 37, 38, 43, 44) were included to identify the association between sleeping outdoors and the pooled prevalence of VL. Seven of the included studies had a significant association with VL. Based on the results of the meta-analysis, it was revealed that the odds for the occurrence of VL infection among people who practiced outdoor sleeping were 3.76 times higher when compared to people who did not practice outdoor sleeping [POR = 3.76; 95% CI: 2.26–5.25]. The association between the presence of VL- infected family member/s and the prevalence of VL was assessed by the included three studies (31, 45, 64). Two of the included studies showed a positive association with the prevalence of VL. The result of this meta-analysis found that the odds of VL infection were 3.6 times higher among people who had VL- infected family member/s compared to those who did not have VL- infected family member/s [POR = 3.59; 95% CI: 1.94–5.24].

The association between the presence of water source/pathway near home and VL infection was determined by two studies (28, 64). The association was positive in both of the studies. The random-effect meta-analysis of this study revealed that the odds of VL infection were 4.29 times higher among people whose home is near to water source/pathway compared to their counterparts [POR = 4.29; 95% CI: 1.89–6.70] (Table 3).

## Discussion

Visceral leishmaniasis is a devastating parasitic disease that has continued to be a global burden to have a substantial impact on health that primarily affects the poorest and most marginalized communities in the world (25, 73). The burden of VL is particularly high in South Asia, East Africa, and the Mediterranean basin. The economic and social consequences of VL are severe, as the disease disproportionately affects individuals who are already vulnerable (10, 25). VL is a major public health problem, particularly in Eastern Africa, where it is considered a neglected tropical disease. The disease is endemic in countries of Eastern Africa like Ethiopia, Kenya, Sudan, and South Sudan, with high transmission rates in rural communities (25, 74, 75). The impact of VL in East Africa is compounded by factors like poverty, poor sanitation, conflict, and displacement (26).

The pooled prevalence of human visceral leishmaniasis in Eastern Africa was 26.16% [95%; CI: 19.96, 32.36%]. However, the report is higher than the pooled prevalence of VL obtained from a global systematic review and meta-analysis done among blood donors (7%) (19) and peoples with human immunodeficiency virus (HIV) (6%) (76). Moreover, the figure is higher than the findings of systematic reviews and meta-analysis pooled prevalence report of human VL in Iran conducted by Rahmanian et al. (3%) (20) and Rostamian et al. (2%) (2). The finding is also higher than the reported pooled prevalence of visceral leishmaniasis in Ethiopia by Haftom et al. (9.44%) (21) and Ayalew Assefa (16%) (22). The high prevalence and the pattern might reflect the lack of and inefficient access to affordable and active drugs, incorrect prescribing, and poor compliance undermine case management and perpetuate anthroponotic infection, and inadequate and unsustainable vector control (77). Other evidence suggests that this trend is continued due to the continuing widespread conflict in Eastern Africa, which destroyed housing and health care infrastructure. This in turn resulted in forced migrations to endemic areas that promote the emergence of VL (78). Moreover, the lack of advancement in diagnostics in field and facility settings, proficient vaccination of the disease, and unaffordability of the new advanced technologies contribute to the pattern to continue (79, 80).

Regarding subgroup analysis, the pooled prevalence of visceral leishmaniasis across the study setting is significantly heterogeneous after a univariate meta-regression. This might be because community-based studies often include a broader and more diverse population, including asymptomatic individuals or those with mild symptoms who might not seek medical care. In contrast, healthcare facility studies typically involve patients already symptomatic and seeking treatment, leading to higher observed prevalence rates. Moreover, community-based studies might use random sampling, while healthcare facility studies often use convenience sampling, which can introduce bias.

The univariate meta-regression analysis also found that the sample size category was a significant factor for the heterogeneity of the pooled prevalence of visceral leishmaniasis in Eastern Africa. This might be due to the statistical power in which the studies with smaller sample sizes often have less statistical power which leads to greater variability in prevalence estimates. This variability can contribute to heterogeneity when these studies are pooled with larger studies. Moreover, the sampling errors might be the reason for the significant heterogeneity of the pooled prevalence as smaller studies are more susceptible to sampling error, where the sample may not accurately represent the broader population. This can result in prevalence estimates that differ significantly from those of larger, more representative studies.

The odds of VL infection were higher among males compared to females. This finding is supported by a systematic review and meta-analysis report in Ethiopia in which males were 67% at higher risk of VL infection as compared to females (21), and a study conducted in India and Nepal which revealed that males were at greater risk of VL infection by a factor of 2.4 as compared to females (81). This might be because, socio-culturally, men are often more exposed to environments where sandflies, the vectors of the disease, are prevalent. This is because men are more likely to engage in outdoor activities such as farming, herding, or sleeping outside, which increases their risk of being bitten by infected sandflies which results in VL infection (82, 83).

The odds of VL infection were higher among people aged below 15 years compared to people aged 15 years and above. This finding is supported by the fact that Eastern Africa accounted for 73% of global VL cases, half of which occurred in children aged under 15 years in 2022 according to the WHO (84). This might be due to people aged less than 15 years being more likely to engage in activities that increase their exposure to sandfly bites, such as playing outdoors, which further elevates their risk. However, contrary to this finding, a cross-sectional study conducted in Southeastern Nepal revealed that the risk of VL infection among people aged ≥15 years was 5.5 times greater as compared to people aged <15 years (4). This inconsistency might be due to the difference in outdoor movement practiced by people under fifteen years and their counterparts. The odds of VL infection were 2.83 times higher among people who had a family of more than five as compared to their counterparts. This finding is supported by a study conducted in Nepal which elucidated that the risk of VL infection among families ≥6 in number was greater by a factor of 4.4 compared to their counterparts (4). Similarly, another study conducted in Nepal evidenced that large size households (>5 persons) were 3.6 times at greater risk of VL infection (85).

The odds of VL infection were higher among people who had termite hills/mounds near their homes compared to their counterparts. This finding is supported by a systematic review that explored the burrows of mammals, caves, crevices, termite hills, and walls with cracks that may serve as breeding sites for sand flies which later increase the transmission and infection of VL (86). The finding is also supported by a study conducted in Ethiopia in which the presence of termite hills was a risk factor for visceral leishmaniasis (87). Moreover, the presence of termite hills is one of the major landscape factors associated with increasing the risk of leishmaniasis infection. The presence of termite hill increases the risk of infection by a factor of 2.4 (88). This might be due to the roles as breeding and resting sites for sandflies, the primary vectors of the disease. This is evidenced by a study conducted in Colombia (89) in which termite hills provide an ideal microhabitat with stable temperature and humidity, which are conducive to the lifecycle of sandflies. These insects thrive in such environments, increasing the likelihood of human-sandfly interactions, especially in rural areas where people live or work near termite mounds. Consequently, individuals in these areas are at a higher risk of being bitten by infected sandflies, leading to a greater incidence of visceral leishmania.

The odds of VL infection were higher among people who had cattle/domestic animals compared to their counterparts. This aligns with findings from a systematic review of South Asian VL (90) and a study in India (91), which indicated that livestock ownership increases the risk of VL infection. In this meta-analysis, it was revealed that the odds for the occurrence of VL infection among people who practiced outdoor sleeping were 3.76 times higher when compared to people who did not practice outdoor sleeping. This finding is supported by a systematic review on VL (86) and a study conducted in India (92) which evidenced that sleeping outside was associated with increased risk of VL. This might be due to the increased exposure to sandflies which are most active during the night and sleeping outdoors increases the likelihood of being bitten by these insects, which can carry the *Leishmania* parasite. Moreover, lack of protective measures such as insecticide-treated bed nets or screens, and environmental factors of the outdoor environments (like vegetation, humidity, and temperature) especially in endemic areas, often provide ideal breeding grounds for and attract sandflies to outdoor sleeping areas which increase the chance of sandfly biting and risk of VL infection in the end.

The odds of VL infection higher among people who had VL- infected family member/s compared to those who did not have VL- infected family member/s. This finding is supported by a systematic review and meta-analysis of factors associated with visceral leishmaniasis in the Americas (93), and a systematic review of environmental and socioeconomic risk factors associated with visceral leishmaniasis (86). Another study conducted in Nepal supported this finding and evidenced that the proximity to previous VL cases was a strong risk factor for VL by 3.79-fold (85). Moreover, this is also congruent with another study conducted in India (92) in which living in the same household with a VL case was associated with a markedly elevated risk of VL. The reason for this might be the shared environmental and behavioral factors within households, such as living conditions and exposure to sandflies (the vector for VL), contribute to the increased risk of the disease.

The odds of VL infection were higher among people whose homes are near water source/pathway compared to their counterparts. This finding is supported by a study in Nepal, which demonstrated that living near water sources, such as ponds, significantly increased the risk of VL infection by a factor of 3.7 (4). Similarly, a study in India also found that proximity to water bodies increased the risk of VL infection (91).

### Limitations of the study

Even though the systematic review and meta-analysis was conducted based on the latest PRISMA guideline, it faced limitations due to the substantial heterogeneity among included studies. The disparity in sample sizes, with some studies involving large participants, while others involved small participants, introduced potential bias and limited the generalizability of findings. Furthermore, the contrasting study settings, with some studies conducted in healthcare facilities and others in community settings, created substantial variability in exposure to risk factors and VL prevalence. This heterogeneity made it challenging to draw robust conclusions about the pooled prevalence of VL and its associated risk factors. Despite these limitations, this review provides valuable insights into the complexities of VL transmission and highlights the need for further research to address the most effective intervention mechanism/s.

## Conclusion

In this study, the recorded pooled prevalence of human visceral leishmaniasis in Eastern Africa was higher (26.16%). Gender, age, family size, presence of termite hill/mound, presence of cattle/domestic animals, outdoor sleeping, presence of VL infected family member/s, and presence of water source/pathway near home were the risk factors significantly associated with human visceral leishmaniasis. While further research is needed to determine the most effective and timely intervention methods for visceral leishmaniasis, this study highlights the urgent need for comprehensive intervention strategies in Eastern Africa. This includes rigorous health education for residents, covering the disease’s cause, transmission, vector, and breeding sites, with strong collaboration between the WHO, government officials, non-governmental organizations (NGOs), and healthcare providers. Additionally, promoting health-seeking behavior for prompt treatment and implementing prevention strategies like avoiding outdoor sleep between dusk and dawn, wearing protective clothing, and using insect repellents are crucial steps toward controlling VL in the region.

## Data Availability

The raw data supporting the conclusions of this article will be made available by the authors, without undue reservation.
