# Supplementary material for: Prevalence of human visceral leishmaniasis and its risk factors in Eastern Africa: a systematic review and meta-analysis
Source: Front Public Health. 2024 Nov 21;12:1488741. doi: 10.3389/fpubh.2024.1488741 (PMC11628699; doi:10.3389/fpubh.2024.1488741)
Supplement: Supplementary file 1 [file Table_1.docx]

**Results of JBI Quality Assessment for Cross-sectional Studies**

| Studies | Criteria | | | | | | | | | |
| --- | --- | --- | --- | --- | --- | --- | --- | --- | --- | --- |
|  | Clear eligibility criteria | Description of study subject and study setting | Valid & reliable method to measure the exposure | Standard criteria used for measurement of the condition | Identification of confounding factors | Development of strategies to deal with confounding factors | Valid and reliable method to measure outcomes | Appropriate statistical analysis | Total score out of 8 | Quality Score (%) |
| Ho et al | No | Yes | Yes | Yes | No | No | Yes | Yes | 5 | 62.5 |
| Kanyina | Yes | Yes | Yes | Yes | Yes | Yes | Yes | Yes | 8 | 100 |
| Ali and Ashford (a) | No | Yes | No | Yes | No | No | Yes | No | 3 | 37.5 |
| Lotukoi | Yes | Yes | Yes | Yes | No | No | No | Yes | 5 | 62.5 |
| Ryan et al | Yes | Yes | Yes | No | No | No | Yes | Yes | 5 | 62.5 |
| Abdalla et al | Yes | Yes | Yes | Yes | No | No | Yes | Yes | 6 | 75 |
| El-Safi et al | Yes | Yes | No | Yes | No | No | Yes | Yes | 5 | 62.5 |
| Hailu et al | No | Yes | No | No | No | No | Yes | No | 2 | 25 |
| Ketema et al | Yes | Yes | Yes | Yes | Yes | Yes | Yes | Yes | 8 | 100 |
| Ibrahim et al | Yes | Yes | No | Yes | No | No | Yes | Yes | 5 | 62.5 |
| Mohamed et al | Yes | Yes | Yes | Yes | Yes | Yes | Yes | Yes | 8 | 100 |
| Odoch and Olobo | Yes | Yes | Yes | Yes | No | No | Yes | Yes | 6 | 75 |
| Abera et al | Yes | Yes | Yes | Yes | No | No | Yes | No | 5 | 62.5 |
| Ayehu et al | Yes | Yes | Yes | Yes | No | Yes | Yes | Yes | 7 | 87.5 |
| Azene et al | Yes | Yes | Yes | Yes | No | No | No | Yes | 5 | 62.5 |
| Bejano et al | Yes | Yes | Yes | Yes | Yes | Yes | Yes | Yes | 8 | 100 |
| Bsrat et al | Yes | Yes | Yes | Yes | Yes | Yes | Yes | Yes | 8 | 100 |
| Custodio et al | Yes | Yes | Yes | Yes | No | Yes | Yes | Yes | 7 | 87.5 |
| Ismail et al | Yes | Yes | Yes | Yes | Yes | Yes | Yes | Yes | 8 | 100 |
| Shiddo et al | No | Yes | No | No | No | No | Yes | No | 2 | 25 |
| Lemma et al | Yes | Yes | Yes | Yes | Yes | Yes | Yes | Yes | 8 | 100 |
| Melkie et al | Yes | Yes | Yes | Yes | Yes | No | Yes | Yes | 7 | 87.5 |
| Tadese et al | Yes | Yes | Yes | Yes | Yes | Yes | Yes | Yes | 8 | 100 |
| Alebie et al | Yes | Yes | Yes | Yes | Yes | Yes | Yes | Yes | 8 | 100 |
| Ali and Ashford (b) | Yes | Yes | Yes | Yes | No | No | Yes | No | 5 | 62.5 |
| Abubakar et al | No | Yes | No | Yes | No | No | Yes | No | 3 | 37.5 |
| Ali et al | Yes | Yes | Yes | Yes | No | No | Yes | No | 5 | 62.5 |
| Bekele et al | Yes | Yes | Yes | Yes | Yes | Yes | Yes | Yes | 8 | 100 |
| van Griensven et al | Yes | Yes | Yes | Yes | Yes | Yes | Yes | Yes | 8 | 100 |
| Abdullahi et al | Yes | Yes | Yes | Yes | No | Yes | Yes | Yes | 7 | 87.5 |
| Sordo et al | No | Yes | No | Yes | No | No | Yes | No | 3 | 37.5 |

**Results of JBI Quality Assessment for Case Control Studies**

| Studies | Criteria | | | | | | | | | | | |
| --- | --- | --- | --- | --- | --- | --- | --- | --- | --- | --- | --- | --- |
|  | Comparability | Case and controls matched properly | Use the same criteria for cases & control | standard, valid and reliable exposure assessment | Exposure measured cases & control similar way | confounding factors identified | strategies for confounding factors stated | Outcomes assessed in a standard, valid and reliable way | Enough exposure period | Appropriate statistical analysis used | Total score out of 10 | Quality Score (%) |
| Dulacha et al | Yes | Yes | Yes | Yes | Yes | Yes | No | Yes | Yes | Yes | 9 | 90 |
| van Dijk et al | Yes | Yes | Yes | Yes | Yes | Yes | No | Yes | Yes | Yes | 9 | 90 |
| Nackers et al | Yes | No | Yes | Yes | Yes | Yes | No | Yes | Yes | Yes | 8 | 80 |
| Bantie et al | Yes | No | Yes | Yes | Yes | No | No | Yes | Yes | Yes | 7 | 70 |
| Yared et al | Yes | Yes | Yes | Yes | Yes | Yes | No | Yes | Yes | Yes | 9 | 90 |
| Kolaczinski et al | No | Yes | Yes | No | Yes | No | No | No | No | Yes | 4 | 40 |

**Results of JBI Quality Assessment for cohort studies**

| Studies | Criteria | | | | | | | | | | | | | |
| --- | --- | --- | --- | --- | --- | --- | --- | --- | --- | --- | --- | --- | --- | --- |
|  | Were the two groups similar & recruited from the same population? | Exposed & unexposed measured similarly | exposure measured in a valid & reliable way | confounding factors identified | strategies for confounding factors stated? | free of the outcome at the start of the study | outcomes measured in a valid and reliable way | follow up time reported & sufficient to be long enough | Complete  follow up | strategies to address incomplete follow up utilized | appropriate statistical analysis used | Total score out of 11 | Quality Score (%) |  |
| Mueller et al | Yes | Yes | Yes | No | No | No | Yes | No | Yes | No | Yes | 6 | 54.5 |  |
| Ayalew and Abere | Yes | Yes | Yes | No | No | No | Yes | No | Yes | No | Yes | 6 | 54.5 |  |
| Gize et al | Yes | Yes | Yes | No | Yes | Yes | Yes | No | Yes | No | Yes | 8 | 72.7 |  |
| Terefe et al | Yes | Yes | Yes | No | No | Yes | Yes | No | Yes | No | Yes | 7 | 63.6 |  |
| Yimer et al | Yes | Yes | Yes | No | Yes | Yes | Yes | Yes | Yes | No | Yes | 9 | 81.8 |  |
| Diro et al | Yes | Yes | Yes | No | No | No | Yes | No | Yes | No | Yes | 6 | 54.5 |  |
| Wondimeneh et al | Yes | Yes | Yes | No | Yes | Yes | Yes | No | Yes | No | Yes | 8 | 72.7 |  |
| Marlet et al | Yes | Yes | Yes | No | No | No | Yes | No | Yes | No | Yes | 6 | 54.5 |  |
